# The relationship between mental health/physical activity and pain/dysfunction in working-age patients with knee osteoarthritis being considered for total knee arthroplasty: a retrospective study

**DOI:** 10.1186/s42836-021-00077-5

**Published:** 2021-07-01

**Authors:** Juyang Jiao, Haozheng Tang, Shutao Zhang, Xinhua Qu, Bing Yue

**Affiliations:** Department of Bone and Joint Surgery, Department of Orthopaedics, Renji Hospital, School of Medicine, Shanghai Jiao Tong University, 145, Shandong Road, Shanghai, 200011 China

**Keywords:** Depression, Pain, Physical activity, Total knee arthroplasty

## Abstract

**Background:**

Increasing total knee arthroplasty (TKA) surgeries are being performed on working-age patients with prominent manifestations of pain and dysfunction. But few studies have explored the risk factors for pain and dysfunction in working-age patients with knee osteoarthritis (KOA) being considered for TKA. Therefore, this study sought to explore the relationship between mental health/physical activity and pain/dysfunction in working-age patients with KOA being considered for TKA.

**Methods:**

This study was a secondary analysis of data derived from a public database, the Work participation In Patients with Osteoarthritis cohort study, which included 152 working-age patients (65 men and 87 women) with KOA planning for TKA. We analyzed preoperative data comprising age, educational level, body mass index (BMI), mental factors (Patient Health Questionnaire-9 [PHQ-9] and the 36-Item Short Form Survey Instrument [SF-36 mental health]), physical activity level, and clinical outcomes (the Western Ontario and McMaster Universities Osteoarthritis Index and SF-36 sub-item score). Multivariate regression analysis was performed to determine risk factors for pain and dysfunction in working-age patients with KOA being considered for TKA.

**Results:**

Women had lower pain, worse function, and higher PHQ-9 scores than men (*p* < 0.001). The depression scores were significantly linearly related to pain and function scores in women after adjusting for age, BMI, educational level, and physical activity (*P* < 0.05), whereas this relation was not observed in men. After adjusting for age, BMI, educational level, and mental factors, exercise time was found to be positively correlated with pain scores in women (*P* < 0.05).

**Conclusions:**

Depression scores and exercise time were significantly correlated with pain and dysfunction in working-age women with KOA being considered for TKA.

## Background

Total knee arthroplasty (TKA) is an effective treatment for advanced knee osteoarthritis (KOA) that is refractory to conservative treatment. Since 2009, cases of TKA in the United States have exceeded 600,000 per year [1]. Among these patients undergoing TKA, 30–45% were working-age individuals. It was projected that, by 2030, the percentage of working-age patients being considered for TKA would be 55% [2]. These patients often have the following characteristics: (1) relatively mild radiological severity and poor preoperative pain and functional scores [3, 4]; (2) more unsatisfactory results, mainly due to postoperative persistent/residue pain, stiffness, and inability to return to previous work [5–7]; and (3) a higher early revision rate [8, 9]. Therefore, the identification of relevant factors for working-age patients with KOA being considered for TKA is conducive to pain management.

Previous studies have reported various risk factors for pain and dysfunction in older patients with KOA [10, 11]. Female sex, obesity/overweight, previous knee injury, and knee malalignment have been reported as moderate to strong risk factors [10]. Heavy-work activities, especially work that involves frequent kneeling and heavy lifting, and several high-impact sports (*e.g.*, football, weight-lifting, and long-distance running) have also been reported to be associated with increased risk for KOA. Weakness of the knee extensor muscle may be a weak risk factor [11]. However, few studies have explored the risk factors for pain and dysfunction in working-age patients with KOA being considered for TKA. These two types of patients are obviously different in their working state and health status and cannot be used for reference. Hence, it is crucial to understand the factors related to pain and functional scores in working-age patients with KOA being considered for TKA.

As such, the purpose of this study was to investigate (1) the relationship between mental health, physical activity, and pain/dysfunction in working-age patients with KOA being considered for TKA and (2) whether there is a gender difference in this relationship.

## Methods

### Source of data and patient selection

Our study was a secondary analysis of data derived from a public database, the Work Participation In Patients with Osteoarthritis cohort study [12]. Overall, 152 (65 men and 87 women) patients with KOA with preoperative employment, being considered for TKA, with a mean age of 55 years (standard deviation (SD) = 5.5, range, 28–63 years) and complete preoperative information, were enrolled. Failure to complete questionnaires because of language difficulties and a previous history of joint surgery were the exclusion criteria.

### Data elements

Preoperative data were mainly obtained by administering multiple questionnaires to the patients. The following preoperative demographic characteristics were collected: general demographic characteristics, patient-related clinical outcomes on pain and function, mental health, and physical activity. General demographics included age, sex, body mass index (BMI), and educational level. Preoperative patient-reported outcomes were primarily based on the Western Ontario and McMaster Universities Arthritis Index (WOMAC) [13], a reliable OA-specific test that comprises three items, namely, pain, stiffness, and physical function, and the 36-Item Short Form Survey Instrument (SF-36), a generic health scale [14]. We calculated the WOMAC scores by converting the scores for each item of the WOMAC into a scale of 0–100 points, with a higher score indicating a better result. For example, the higher the pain score, the lower the pain. Pain was reflected by WOMAC pain and SF-36 pain scales; joint function measures included WOMAC physical function, SF-36 physical function, and SF-36 physical role function scale. Mental and psychological activities were assessed using the SF-36 mental health scale and Patient Health Questionnaire-9 (PHQ-9) [15]. PHQ-9 mainly reflects depressive symptoms, whereas the SF-36 mental health scale aptly hints at general psychological distress, which has been validated as a standard measure for distress. Habitual physical activity was assessed through the Short Questionnaire to Assess Health-Enhancing Physical Activity (SQUASH) questionnaire, which mainly investigates the time, frequency, and extent of physical activity of different subjects per week [16], and includes many questions on specific physical activity items, such as commuting, housework, sports, and leisure time. In addition, social characteristics are reflected by the SF-36 social function scale and actual working hours.

### Statistical analysis

We separately performed analyses in two groups: working-age women and men. Continuous variables are described as mean ± SD, and categorical variables are represented as number and proportion. Statistical assessment of categorical variables was accomplished using the chi-square test. Additionally, normally distributed continuous variables were compared using one-way analysis of variance and skewed continuous variables using the Kruskal-Wallis test.

Using unadjusted and multivariate-adjusted logistic regression analyses, we determined the regression coefficient and corresponding 95% confidence interval (CI) for associations between mental factors per 10 points in the SF-36 mental health scale and depression PHQ-9 scores and preoperative clinical outcome scores containing pain and joint function in each group. The crude model was not adjusted for any variables. Multivariate-adjusted model was adjusted for age, BMI, educational level, and physical activity. Likewise, we analyzed the effect of physical activity per 10 actual working hours per week and per 100 min of cycling, sports, total physical activity, and different physical activity levels on preoperative clinical outcome scores. All aforementioned statistical analyses were performed using the R package (http://www.r-project.org). A two-sided *P*-value < 0.05 was considered to be statistically significant.

## Results

### Comparison of baseline characteristics between working-age men and women with KOA being considered for TKA

General characteristics of working-age TKA patients are presented in Table 1. Women reported worse clinical outcome scores for WOMAC pain, SF-36 pain, WOMAC physical function, and SF-36 physical function (*P* < 0.05) than men. No significant differences were noted between men and women in age, BMI, SF-36 physical function, and SF-36 emotional role function scores. The mean BMI of men and women in this study was higher than the upper limit of normal values recommended by the World Health Organization. Women had a slightly higher educational level than men (*P* = 0.041). Regarding mental factors, the mean PHQ-9 depression (*P* < 0.001) and SF-36 mental health (*P* < 0.001) scores were significantly higher in women than in men. Concerning the physical activity level, men spent more time working, but less time doing housework, than women (*P* < 0.001). However, there was no significant difference between them in terms of the time for total physical activity, cycling, gardening, commuting, leisure, and sports.

**Table 1:** General characteristics of working-age TKA patients based on gender

Characteristics	Male patients	Female patients	***P***-value
No	65	87	
Age (no. (%))			0.431
0–45 years	2 (3.08%)	7 (8.05%)	
46–55 years	25 (38.46%)	33 (37.93%)	
56–64 years	38 (58.46%)	47 (54.02%)	
Educational Level (%)			0.041
Low	29 (45.31%)	22 (25.58%)	
Secondary	24 (37.50%)	44 (51.16%)	
High	11 (17.19%)	20 (23.26%)	
BMI (mean)	28.24 ± 3.19	31.08 ± 5.93	0.073
Depression PHQ-9 Score (mean)	2.86 ± 3.38	4.99 ± 3.91	< 0.001
WOMAC Pain (mean)	49.80 ± 21.65	35.92 ± 21.21	< 0.001
WOMAC Physical function (mean)	56.25 ± 17.41	41.47 ± 16.96	< 0.001
SF-36 Pain (mean)	42.98 ± 20.30	34.90 ± 17.65	0.01
SF-36 Mental health (mean)	83.06 ± 14.60	74.76 ± 13.78	< 0.001
SF-36 Vitality (mean)	68.20 ± 18.31	54.12 ± 18.10	< 0.001
SF-36 Social function (mean)	76.37 ± 22.40	64.08 ± 29.21	0.006
SF-36 Physical function (mean)	39.28 ± 17.43	25.87 ± 12.85	< 0.001
SF-36 Physical role function (no (%))			0.076
0	22 (34.38%)	45 (51.72%)	
25	10 (15.62%)	13 (14.94%)	
50	10 (15.62%)	9 (10.34%)	
75	6 (9.38%)	11 (12.64%)	
100	16 (25.00%)	9 (10.34%)	
SF-36 Emotional role function (no (%))			0.496
0	9 (14.29%)	18 (20.69%)	
33	5 (7.94%)	6 (6.90%)	
67	3 (4.76%)	8 (9.20%)	
100	46 (73.02%)	55 (63.22%)	
Real working hours / week (mean)	39.15 ± 13.56	25.14 ± 12.26	< 0.001
Cycling activities (mean minutes / week)	95.68 ± 122.16	144.09 ± 174.95	0.07
Gardening activities (mean minutes / week)	198.81 ± 763.43	88.66 ± 147.00	0.204
Household activities (mean minutes / week)	301.21 ± 428.92	1153.00 ± 958.75	< 0.001
Light	284.44 ± 424.87	986.76 ± 781.20	< 0.001
Vigorous	16.77 ± 41.88	166.24 ± 367.33	0.002
Commuting activites (mean minutes / week)	204.95 ± 597.07	134.81 ± 569.07	0.472
Sports activities (mean minutes / week)	68.47 ± 153.33	79.88 ± 137.55	0.644
Leisuretime activities (mean minutes / week)	654.83 ± 956.99	439.45 ± 377.88	0.067
Work activities (mean minutes / week)	1815.00 ± 955.53	1314.42 ± 667.22	< 0.001
Total minutes (mean minutes / week)	2909.02 ± 1431.79	3004.70 ± 1278.75	0.667
Light	1843.55 ± 1271.08	2149.90 ± 1062.44	0.111
Moderate	837.66 ± 1059.58	681.56 ± 753.73	0.293
Vigorous	227.81 ± 755.75	173.24 ± 258.69	0.534

*BMI* body mass index, *no*. number, *SF-36* 36-Item Short Form Survey Instrument, *TKA* total knee arthroplasty, *WOMAC* Western Ontario and McMaster Universities Arthritis Index

### Effects of mental factors on preoperative pain and function in working-age patients with KOA being considered for TKA

First, we studied the effects of mental and psychological factors on preoperative pain in working-age TKA patients (Table 2**)**. For women, the PHQ-9 depression scores were negatively correlated with SF-36 pain (*P* = 0.0008) and WOMAC pain (*P* < 0.001), respectively. The associations were still significant after adjusting for age, BMI, and physical activity. No relationship was noted between SF-36 mental health and pain in the non-adjusted and adjusted models in women. Although the SF-36 mental health and PHQ-9 depression scores were significantly correlated with SF-36 and WOMAC pain in men, the correlations were not significant in the adjusted model.

**Table 2:** Relationship between mental factors and clinical outcome scores based on gender

Exposure	Male patients				Female patients			
	B	***P***-value	adjusted B^**a**^	***P***-value	B	***P***-value	adjusted B^**a**^	***P***-value
SF-36 Pain								
Per 10 points in SF-36 mental health	4.27 (0.74, 7.81)	0.0210	0.83 (−3.67, 5.32)	0.7209	3.20 (0.00, 6.40)	0.0532	3.38 (−0.15, 6.91)	0.0660
Depression PHQ-9 score	−2.01 (− 3.54, − 0.49)	0.0121	− 0.97 (− 2.98, 1.03)	0.3485	−1.92 (− 3.00, − 0.84)	0.0008	− 1.71 (− 2.98, − 0.44)	0.0107
WOMAC Pain								
Per 10 points in SF-36 mental health	3.56 (0.22, 6.91)	0.0410	1.18 (− 3.37, 5.72)	0.6152	3.02 (0.37, 5.67)	0.0280	1.69 (−1.36, 4.73)	0.2823
Depression PHQ-9 score	−1.64 (−3.09, − 0.20)	0.0296	− 1.20 (− 3.22, 0.81)	0.2508	−1.98 (− 2.84, − 1.12)	< 0.0001	−1.15 (− 2.24, − 0.05)	0.0451
SF-36 Physical Functioning								
Per 10 points in SF-36 mental health	1.88 (−1.05, 4.82)	0.2132	1.51 (− 2.38, 5.39)	0.4526	3.00 (1.12, 4.87)	0.0024	1.99 (− 0.13, 4.11)	0.0712
Depression PHQ-9 score	− 0.94 (− 2.19, 0.32)	0.1479	−1.01 (− 2.73, 0.70)	0.2546	− 1.44 (− 2.07, − 0.82)	< 0.0001	−1.16 (− 1.90, − 0.41)	0.0037
SF-36 Physical Role Functioning								
Per 10 points in SF-36 Mental health	0.34 (0.08, 0.60)	0.0142	0.28 (− 0.12, 0.68)	0.1782	0.39 (0.18, 0.59)	0.0004	0.40 (0.16, 0.63)	0.0017
Depression PHQ-9 Score	− 0.15 (− 0.26, − 0.03)	0.0128	− 0.10 (− 0.19, − 0.01)	0.2621	− 0.14 (− 0.21, − 0.07)	0.0002	− 0.10 (− 0.19, − 0.01)	0.0342
WOMAC Physical Functioning								
Per 10 points in SF-36 mental health	3.00 (−0.24, 6.24)	0.0752	1.16 (− 3.54, 5.85)	0.6339	2.38 (−0.32, 5.09)	0.0887	2.20 (−0.70, 5.10)	0.1442
Depression PHQ-9 score	− 1.18 (− 2.56, 0.20)	0.1013	− 1.11 (− 3.26, 1.05)	0.3239	− 1.84 (− 2.72, − 0.97)	< 0.0001	− 1.46 (− 2.48, − 0.45)	0.0067

*B* beta coefficient, *OR* odds ratio, *PHQ-9* Patient Health Questionnaire, *SF-36* 36-Item Short Form Survey Instrument, *WOMAC* Western Ontario and McMaster Universities Arthritis Index

a: adjusted by age, BMI, educational levels and physical activity

As for dysfunction, univariate analysis revealed the association of mental factors with function scores, except for SF-36 and WOMAC physical function scores, in men. In the adjusted model, depression scores were still negatively correlated with the SF-36 physical function scores (OR -1.16; 95% CI, − 1.90 to − 0.41; *P* = 0.0037), SF-36 physical role function (OR -0.10; 95% CI, − 0.19 to − 0.01; *P* = 0.0342), and WOMAC physical function (OR –1.46; 95% CI, − 2.48 to − 0.45; *P* = 0.0067) in women, whereas SF-36 mental health and depression scores were not associated with dysfunction in men.

### Effects of physical activity on preoperative pain and function in working-age patients with KOA being considered for TKA

As shown in Table 3, univariate analysis showed that sports time per week was positively correlated with SF-36 pain in women (*P* = 0.0432), indicating that upon adding every 100 min of sports per week, SF-36 pain would improve by 2.88 points. After ruling out various confounding factors, including age, BMI, educational level, and mental and psychological factors, this relationship still existed (*P* = 0.0164). Unexpectedly, in multiple regression analysis, the SF-36 pain scores in women were also positively correlated with cycling (*P* = 0.0282) and leisure time (*P* = 0.0413). WOMAC pain in women was negatively correlated with actual working hours (*P* = 0.0235). Total physical activity had no significant effect on pain in young men and women. There was also no significant association between pain and individual activity in men.

**Table 3:** Relationship between physical activity and clinical outcome scores based on gender

Exposure	Male				Female			
	B	***P***-value	adjusted B^**a**^	***P***-value	B	***P***-value	adjusted B^**a**^	***P***-value
WOMAC Pain								
Cycling (*per 100 min/week)	−3.19 (− 7.79, 1.40)	0.1779	− 3.46 (− 7.80, 0.88)	0.1258	1.82 (− 0.85, 4.48)	0.1855	1.87 (− 0.84, 4.59)	0.1802
Sports (*100 min/week)	− 1.03 (− 4.74, 2.68)	0.5875	0.18 (− 3.51, 3.86)	0.9261	2.26 (− 1.13, 5.66)	0.1951	2.49 (− 0.89, 5.88)	0.1531
Real working hours (*10 h/week)	1.24 (− 2.92, 5.39)	0.5617	3.28 (− 1.17, 7.72)	0.1553	− 4.21 (− 7.93, − 0.49)	0.0293	− 4.55 (− 8.40, − 0.70)	0.0235
Total minutes (*100 min/week)	−0.02 (− 0.40, 0.36)	0.9157	0.05 (− 0.34, 0.44)	0.8125	−0.17 (− 0.52, 0.18)	0.3455	0.01 (− 0.39, 0.40)	0.9778
Light intensity minutes	−0.10 (− 0.52, 0.32)	0.6437	0.26 (− 0.23, 0.75)	0.3027	− 0.20 (− 0.63, 0.22)	0.3544	− 0.21 (− 0.65, 0.22)	0.3406
Moderate intensity minutes	− 0.06 (− 0.57, 0.45)	0.8206	− 0.28 (− 0.82, 0.25)	0.3028	− 0.22 (− 0.82, 0.38)	0.4676	0.58 (− 0.15, 1.30)	0.1235
Vigorous intensity minutes	0.33 (− 0.38, 1.04)	0.3694	0.11 (− 0.58, 0.79)	0.7621	1.13 (− 0.61, 2.86)	0.2068	0.54 (−1.90, 2.97)	0.6675
SF-36 Pain								
Cycling (*100 min/week)	−0.85 (− 5.21, 3.51)	0.7040	− 0.25 (− 4.00, 3.50)	0.8963	1.88 (− 0.30, 4.06)	0.0950	2.13 (0.27, 4.00)	0.0282
Sports (*100 min/week)	−0.97 (− 4.44, 2.50)	0.5852	− 0.70 (− 3.79, 2.39)	0.6610	2.88 (0.13, 5.63)	0.0432	2.91 (0.59, 5.22)	0.0164
Leisure-time (*100 min/week)	−0.17 (− 0.72, 0.39)	0.5626	−0.43 (− 0.88, 0.02)	0.0689	0.86 (− 0.15, 1.86)	0.1007	0.90 (0.05, 1.76)	0.0413
Total minutes (*100 min/week)	0.08 (− 0.27, 0.44)	0.6416	0.06 (− 0.28, 0.40)	0.7250	0.08 (− 0.22, 0.37)	0.6121	0.18 (− 0.10, 0.46)	0.2073
Light intensity minutes	0.13 (− 0.26, 0.53)	0.5093	0.42 (0.00, 0.83)	0.0550	0.19 (− 0.16, 0.54)	0.2934	0.18 (− 0.13, 0.49)	0.2615
Moderate intensity minutes	−0.08 (− 0.55, 0.40)	0.7531	− 0.23 (− 0.69, 0.23)	0.3352	− 0.39 (− 0.89, 0.10)	0.1262	−0.03 (− 0.55, 0.50)	0.9133
Vigorous intensity minutes	0.07 (− 0.59, 0.74)	0.8304	−0.23 (− 0.82, 0.36)	0.4445	1.97 (0.57, 3.37)	0.0072	1.61 (−0.09, 3.31)	0.0677
SF-36 Physical Functioning								
Cycling (*100 min/week)	−0.63 (− 4.11, 2.86)	0.7249	0.46 (− 3.10, 4.02)	0.8002	− 0.23 (− 1.78, 1.32)	0.7688	0.16 (−1.25, 1.57)	0.8208
Sports (*100 min/week)	−0.80 (− 3.57, 1.98)	0.5759	−1.11 (− 4.03, 1.81)	0.4615	− 0.82 (− 2.79, 1.15)	0.4169	− 0.63 (− 2.39, 1.12)	0.4812
Total minutes (*100 min/week)	0.08 (− 0.23, 0.38)	0.6245	0.04 (− 0.28, 0.36)	0.8062	−0.18 (− 0.39, 0.03)	0.0984	− 0.12 (− 0.33, 0.10)	0.2924
Light intensity minutes	0.12 (− 0.22, 0.46)	0.4879	0.28 (− 0.11, 0.68)	0.1697	− 0.18 (− 0.44, 0.08)	0.1702	− 0.25 (− 0.48, − 0.02)	0.0398
Moderate intensity minutes	− 0.15 (− 0.56, 0.26)	0.4681	−0.28 (− 0.71, 0.16)	0.2168	−0.21 (− 0.57, 0.15)	0.2593	0.33 (− 0.06, 0.72)	0.1017
Vigorous intensity minutes	0.23 (−0.34, 0.80)	0.4359	0.03 (−0.53, 0.59)	0.9126	0.42 (−0.64, 1.49)	0.4363	−0.34 (− 1.66, 0.98)	0.6169
SF-36 Physical Role Functioning								
Cycling (*100 min/week)	−0.06 (− 0.40, 0.29)	0.7483	0.00 (− 0.32, 0.31)	0.9861	0.03 (− 0.15, 0.21)	0.7290	0.05 (− 0.12, 0.22)	0.5479
Sports (*100 min/week)	−0.14 (− 0.42, 0.13)	0.3046	−0.16 (− 0.41, 0.10)	0.2390	0.20 (− 0.03, 0.43)	0.0871	0.27 (0.07, 0.47)	0.0093
Total minutes (*100 min/week)	0.00 (− 0.03, 0.03)	0.9961	−0.01 (− 0.04, 0.02)	0.5420	0.00 (− 0.02, 0.03)	0.7811	0.02 (− 0.01, 0.04)	0.1382
Light intensity minutes	0.00 (−0.03, 0.03)	0.9558	0.02 (−0.02, 0.05)	0.4028	0.03 (0.00, 0.06)	0.0326	0.04 (0.01, 0.06)	0.0082
Moderate intensity minutes	−0.02 (− 0.05, 0.02)	0.3863	− 0.04 (− 0.08, − 0.01)	0.0214	−0.06 (− 0.10, − 0.02)	0.0062	−0.03 (− 0.08, 0.02)	0.2056
Vigorous intensity minutes	0.04 (−0.02, 0.09)	0.1923	0.02 (−0.03, 0.06)	0.5303	0.03 (−0.09, 0.15)	0.6080	−0.10 (− 0.25, 0.06)	0.2148
WOMAC Physical Functioning								
Cycling (*100 min/week)	−2.81 (− 7.02, 1.40)	0.1979	− 2.42 (− 6.70, 1.86)	0.2750	1.77 (− 0.36, 3.90)	0.1079	1.89 (− 0.04, 3.82)	0.0595
Sports (*100 min/week)	−0.78 (− 3.73, 2.17)	0.6050	−0.44 (− 3.48, 2.60)	0.7778	2.74 (0.05, 5.42)	0.0494	2.80 (0.45, 5.15)	0.0227
Total minutes (*100 min/week)	0.03 (− 0.29, 0.35)	0.8540	−0.08 (− 0.41, 0.24)	0.6292	0.06 (− 0.23, 0.36)	0.6739	0.24 (− 0.05, 0.53)	0.1074
Light intensity minutes	−0.14 (− 0.52, 0.23)	0.4482	−0.01 (− 0.46, 0.43)	0.9521	0.17 (− 0.17, 0.52)	0.3351	0.13 (− 0.19, 0.45)	0.4401
Moderate intensity minutes	0.20 (−0.22, 0.62)	0.3450	−0.03 (− 0.50, 0.44)	0.9104	−0.26 (− 0.76, 0.24)	0.3130	0.38 (− 0.15, 0.92)	0.1632
Vigorous intensity minutes	0.07 (−0.52, 0.65)	0.8257	−0.18 (− 0.75, 0.38)	0.5282	0.69 (− 0.71, 2.10)	0.3371	0.82 (− 1.04, 2.69)	0.3905

*B* beta coefficient, *BMI* body mass index, *OR* odds ratio, *SF-36* 36-Item Short Form Survey Instrument, *WOMAC* Western Ontario and McMaster Universities Arthritis Index

*: multiply; a: adjusted by age, BMI, educational levels and mental factors

Concerning preoperative function, the multiple regression analysis results suggested that exercise in women was positively correlated with the SF-36 physical role function (*P* = 0.0093) and WOMAC function (*P* = 0.0227). Total physical activity was not related to dysfunction in both men and women. For men, no significant correlation was found between individual activity and joint dysfunction.

## Discussion

To our knowledge, this is the first study to analyze the risk factors for pain and dysfunction in working-age patients with KOA being considered for TKA. The results revealed that women had lower self-reported pain and function scores than men, and the PHQ-9 depression score was significantly associated with pain and function in women after adjusting for age, BMI, educational level, and physical activity. Furthermore, total physical activity did not correlate with pain and dysfunction in working-age patients. In terms of individual activity, exercise time was associated with pain and function in working-age women.

Owing to the growing prevalance of obesity and the aging population, there has been a gradual increase in the number of cases of KOA, and advanced KOA is occurring in working-age individuals [17]. Pain is one of the most prominent clinical manifestations of KOA in working-age patients, and is often a crucial indication for surgeons to consider TKA. Pain mechanisms associated with knee OA are complex and need to be explained in a biological-psychological-social mode. Local structural changes in the knee joint, such as synovitis, bone marrow lesions, osteophyte formation, and muscle strength changes, can cause or induce pain, and long-term pain can be affected by psychological-social conditions [18]. Our results showed that the preoperative pain, function, and depression scores were significantly worse in women than in men. This finding is consistent with the results of some previous studies [3, 19]. James* et al*. [19] found that women aged < 55 years (74%) accounted for the majority of young women undergoing TKA. Women had a higher BMI and lower WOMAC function and University of California of Los Angeles (UCLA) activity scores than young men. In their study, in addition to cases of OA, there were cases of other diseases, such as inflammatory joint disease and osteonecrosis. Haynes *et al*. [3] found that younger patients with TKA had fewer imaging findings (less cartilage damage and milder degree of OA) than older patients, but their symptoms (UCLA activity, SF-12, WOMAC scores) were more substantial than those of older patients. Among their patients, women had lower WOMAC pain and function scores than men. This phenomenon could be attributed to sex differences in the nociceptive pathways or physiology and in the cognitive or emotional appraisal of pain [20]. In the biological sphere, differences in hormones, such as estrogen, and the endogenous opioid system, which differs between sexes, influence pain control and psychological changes. From a psychosocial perspective, research supports the cultural stereotype that women are more willing to report pain and that men and women utilize different coping strategies when in pain [21]. Pain catastrophizing mediated the relationship between sex- and pain-related outcomes (pain, disability, and pain behaviors) in KOA [22]. Functional differences in KOA subjects may, in part, be due to known differences in quadriceps muscle strength between women and men [23]. Other possible mechanisms may be complex, and further research on these issues is critically needed.

Regarding the effect of mental and psychological factors on the pain and function of working-age patients with TKA, we found that depression scores were negatively correlated with pain and dysfunction in women. Previous studies have reported the close association of mental and psychological activities with pain [24, 25]. The prevalence of anxiety and depression in patients with TKA is increasing annually. The overall prevalence of depression and anxiety in patients undergoing primary TKA in the United States has been reported to be 12–26% [26, 27]. Patients undergoing TKA who have depression and anxiety tended to be younger, with the peak age ranging from 55 to 64 years [28]. Psychological disorders are strongly associated with the prognosis of TKA, and various complications and pain-related symptoms are more likely to occur [28]. Patients with preoperative anxiety and depression reported worse outcomes and were more likely to be dissatisfied with TKA than patients without anxiety and depression [29]. This relationship was also validated in systematic reviews [30, 31]. Lingard *et al*. [32] reported lower preoperative WOMAC pain and function scores in patients with distress (low SF-36 mental health score). Some clues about the relationship between depression and function have been reported. A previous study evaluated the direct effect of depressive symptomology on physical performance, and the indirect effect, mediated by pain severity and dependent on the participant’s number of depressive symptoms, in individuals with radiographic KOA [33]. Those authors found that pain severity mediated approximately one-fifth of the association between depressive symptoms and physical performance in KOA. Apathy and inertia arising from depressive symptomology may also play an important role in this relationship. An interesting recent study on individuals with or at risk for symptomatic KOA identified the following four subtypes of depression: asymptomatic, catatonic, anhedonic, and melancholic. Anhedonic and melancholic depression subtypes were suggested to be risk factors for increased pain and disability in individuals who developed and experienced symptomatic KOA [34]. Our results suggest that depression scores in working-age women were significantly correlated with preoperative pain and function, and this association was not reported in working-age men. This interesting phenomenon is in line with our clinical observation. As research has developed, chronic pain in OA has been reported to be mainly related to inflammatory reactions and neural mechanisms [35]. Pain sensitization occurs when long-term pain stimuli lead to pathological remodeling of the central nervous system, making it more difficult to control the progression of painful diseases. When central sensitization reaches an irreversible stage, some drugs that can reduce the degree of pain sensitization can be considered [36]. Duloxetine, a selective serotonin-norepinephrine reuptake inhibitor that was previously used to treat mood disorders, such as depression and anxiety, is currently approved by the Food and Drug Administration for the treatment of chronic skeletal muscle pain, including OA [37]. Some multi-center randomized controlled trials (RCTs) have demonstrated the effectiveness of duloxetine in relieving pain and improving function in OA [38, 39]. Consequently, for young patients who are considered for TKA, especially women, the patient’s mental state, in particular depression, anxiety, and energy, should be comprehensively evaluated when considering the optimal timing of surgery, and appropriate anti-anxiety or anti-depression drugs should be prescribed. These treatments may alleviate the pain and improve the function of such patients.

In terms of the effect of physical activity on the pain and function of working-age patients with KOA being considered for TKA, we found no significant correlation between weekly physical activity time and pain or function, regardless of gender. However, concerning a specific physical activity, we found that the pain in women was significantly positively correlated with exercise time. At the same time, joint function and exercise time were positively correlated in women. In fact, the relationship between exercise and development of OA remains controversial. It has been thought that due to the wear and tear of the hyaline cartilage in weight-bearing joints, increased physical activity may induce or aggravate cartilage damage and lead to OA. Currently, most studies support the notion that physical activity is not associated with the development of symptomatic knee OA [40]. Early Framingham cohort studies suggested that daily habitual physical activity is not associated with KOA [41]. Mork *et al*. [42] analyzed baseline data of 15,191 women and 14,766 men without pain or injury in the Norwegian HUNT study, and symptomatic OA was evaluated after 11 years of follow-up. They found that physical activity was not associated with OA; high BMI increased the risk of KOA, but physical activity did not increase the risk of KOA at any BMI level. Toivanen *et al*. [43] investigated a sample of 8000 Finnish individuals. Twenty-two years later, 823 patients without KOA were re-examined, and it was found that regular leisure physical activity reduced the incidence of clinically diagnosed KOA. Exercise can affect joint function and reduce pain by lubricating the joints or improving muscle strength. A systematic review containing multiple RCTs examined the effect of exercise on articular cartilage in patients with KOA or the risk of KOA, and the results showed that knee weight-bearing activity was beneficial for articular cartilage in patients with KOA or at risk of KOA [44]. As a result, for working-age OA patients considered for TKA, especially overweight and obese women, increasing exercise appropriately may improve joint function and reduce pain.

This study has some strengths. First, we analyzed general characteristics, physical activity, and mental-psychological factors. By adjusting some of these variables to study the effect of other factors on pain and function, we found that the factors were more aligned with a modern biological-psychological-social mode. Second, we used two scoring methods (WOMAC and SF-36) to assess the pain and function of KOA, increasing the credibility and accuracy of the study. Nevertheless, it is undeniable that this study has some shortcomings. First, this was a retrospective study that could only explore correlations but could not explain the causal relationship between them. Second, the number of patients was small. Hopefully, a larger sample or population will be available in the future to conduct a more comprehensive analysis and assessment of the risk factors associated with working-age patients who require TKA. Third, some confounding factors, such as baseline arthritis grade, previous injuries, and other anatomic factors, might have affected the results. We will evaluate this in a future prospective study.

## Conclusions

Among working-age patients with KOA being considered for TKA, there are large differences in preoperative pain and function between men and women. Depression scores and exercise time are significantly correlated with preoperative pain and function in working-age women with KOA. The study highlights the importance of psychological assessment and exercise prescription in patients with KOA, especially working-age women, before considering TKA. A comprehensive assessment of the patient’s mental status and physical activity is favorable for the management of preoperative symptoms and can have a positive impact on postoperative recovery.

## Data Availability

The datasets generated and/or analyzed during the current study are available in the Dryad database (url: http://datadryad.org/review?doi=doi:10.5061/dryad.kc260) [12].

## Abbreviations

*BMI*: Body mass index
*CI*: Confidence interval
*KOA*: Knee osteoarthritis
*OA*: Osteoarthritis
*PHQ-9*: Patient Health Questionnaire-9
*RCT*: Randomized controlled trial
*SF-36*: 36-Item Short Form Survey Instrument
SD: Standard deviation
*SQUASH*: Short Questionnaire to Assess Health-Enhancing Physical Activity
*TKA*: Total knee arthroplasty
*OR*: Odds ratio
*UCLA*: University of California, Los Angeles
*vs*.: *Versus*
*WOMAC*: Western Ontario and McMaster Universities Arthritis Index
